# Research on Bacterial Diversity and Antibiotic Resistance in the Dairy Farm Environment in a Part of Shandong Province

**DOI:** 10.3390/ani14010160

**Published:** 2024-01-03

**Authors:** Yuehui Cui, Kaimin Song, Xiaoting Liu, Huiling Xu, Xiaozhou Wang, Guodong Cheng, Pimiao Zheng, Jianzhu Liu

**Affiliations:** 1 College of Veterinary Medicine, Shandong Agricultural University, Tai’an 271018, China; 2 Research Center for Animal Disease Control Engineering, Shandong Agricultural University, Tai’an 271018, China

**Keywords:** intensive dairy farming, environmental microorganisms, antibiotic resistance, antibiotic resistance gene

## Abstract

**Simple Summary:**

In intensive farming, antimicrobials are heavily used for the prevention and treatment of animal diseases. However, the improper use of antimicrobials has led to the widespread presence of antimicrobial-resistant bacteria and antimicrobial-resistant genes in the environment. In this study, five dairy farms located in Shandong Province were selected, and a total of 223 isolates were collected from various environmental locations within each farm (bedding, sports field, and milking parlor). The most frequently detected bacteria were Fusobacterium and Escherichia. The majority of bacteria displayed resistance to multiple antibiotics. The sulfonamide resistance gene *sul1* showed the highest detection rate, which corresponded to the sulfadiazine resistance phenotype. Doxycycline and levofloxacin demonstrated the most effective antibacterial properties. In conclusion, understanding the microbial species present and their antimicrobial resistance profiles aids in focusing efforts toward sustainable antimicrobial use and safeguarding human health.

**Abstract:**

Antimicrobials are extensively utilized in dairy farms to prevent and control diseases in cattle. However, their use contributes to the emergence of antimicrobial-resistant bacteria (ARB) and antimicrobial-resistant genes (ARG), and these can be transmitted to the environment. Regular monitoring of antimicrobial resistance (AMR) is crucial for implementing effective mitigation strategies. This research aimed to assess the environmental microbial species present on dairy farms in Shandong Province and characterize the antimicrobial resistance profiles of the isolates. Five dairy farms located in Shandong Province were selected, representing the prevalent large-scale farming patterns in the area. Sampling took place from April to June 2022, with a total of 223 isolates collected from various environmental locations within each farm (bedding, sports field, and milking parlor). Matrix-assisted laser desorption/ionization—time-of-flight mass spectrometry (MALDI-TOF MS) was employed to identify the species of the clinical isolates. The main pathogens isolated were *Aerococcus viridans* (5.38%, *n* = 12), *Corynebacterium xerosis* (4.93%, *n* = 11), and *Acinetobacter lwoffii* (4.03%, *n* = 9). Among the bacterial isolates, resistance to lincomycin was highest at 91%, and 88% were resistant to sulfadiazine. Antimicrobial resistance genes were detected in only a small proportion of the isolates, the most common of which was *sul1*. These findings highlight the necessity for careful evaluation of antimicrobial usage in maintaining their effectiveness in human medicine. Understanding the microbial species present and their antimicrobial resistance profiles aids in focusing efforts toward sustainable antimicrobial use and safeguarding human health.

## 1. Introduction

It is well recognized that the wide and inappropriate use of antimicrobials is closely related to the emergence and spread of antimicrobial-resistant bacteria. Excessive abuse of antibiotics in the fields of healthcare and animal husbandry and insufficient attention to drug-resistant bacteria (ARB) have emerged in large numbers in the last hundred years, threatening human society. Different classes of antimicrobials are used to treat disease in both humans and animals, and dairy farming is one of the most significant users of antimicrobials [1,2,3]. On dairy farms, antimicrobials are used for a variety of therapeutic (e.g., mastitis, metritis, respiratory disease, and foot disease) and prophylactic (e.g., preventive disinfection, medicated milk replacer in calves) purposes. For now, antimicrobials used in dairy farms mainly include third-generation cephalosporins (TGCs), tetracyclines (TETs), fluoroquinolones, and macrolides [1,4,5]. These antimicrobials are administered to dairy cattle parenterally, orally, or through intramammary infusion (IMMI) [6]. According to research predictions, if the increasing drug-resistant status quo is not effectively improved, the problem of AMR will become increasingly serious, and by 2050, 10 million people will lose their lives globally due to ineffective treatment of ARB infections, resulting in a global economic loss of about 2–3.5% [7]. A recent pan-European study of the antimicrobial susceptibility of milk pathogens causing clinical mastitis in dairy cattle showed a high proportion of *Staphylococcus* resistant to penicillin and *Streptococcus* resistant to erythromycin and tetracycline [8]. Data from China were lacking in these studies.

According to the report, China’s agricultural system has converted from traditional free-range farming to intensive livestock production [9], and China is the largest consumer of veterinary antibiotics, accounting for 45% of global usage in 2017, based on 41,967 tonnes of usage reported by the Chinese Ministry of Agriculture [10]. And the consumption level is more than 200 mg/PCU [11]. The application of antibiotics to prevent and improve the health of growing animals is still an essential part of animal husbandry. Globally, animal farming accounted for 73% of total antimicrobial use in 2017 [12]. In the same year, the Chinese government launched an action plan to curb the antimicrobial resistance of animal-derived bacteria and control the abuse of antimicrobials in animal husbandry. To maintain the effectiveness of antimicrobials that are essential in human medicine, the Chinese government follows the guidelines of the World Health Organization (WHO) to completely ban the use of antimicrobials as feed additives from 2020 in order to reduce the development of bacterial resistance.

Antibiotic resistance leads to delays in the effect of appropriate antibiotic therapy and increases morbidity and mortality. According to a 2019 report from the Centers for Disease Control and Prevention (CDC) in the United States, over 2.8 million cases of ARB infection occur each year, resulting in approximately 35,000 deaths. AR is the genetic ability of a microorganism to survive in an environment of high antibiotic concentrations, and it is often quantified by measuring the minimum inhibitory concentration (MIC) of a particular antibiotic at which ARBs can grow and multiply. In recent years, humans have detected new antimicrobial resistance genes in various regions of the world and found that these ARGs are spreading rapidly around the world, attracting unprecedented attention from human society [13,14,15]. According to studies, ARB and ARGs can be excreted from feces into the soil, and can also be circulated among hospitals, pharmaceutical factories, sewage treatment plants, and animal farms, which has attracted the attention of experts in the field of public health [16,17]. Hu et al. quantified antibiotic contamination in the Yangzi River Basin and concluded that it is closely related to the use of veterinary antibiotics, and thus the use of antibiotics in animal production must be better regulated and animal feces should be properly disposed of [18]. Many governments are currently working with the World Health Organization and the United Nations (UN) to prevent and reduce the development of antimicrobial resistance. It is a battle against time.

In this study, environmental samples were collected from beddings, sports fields, and milking parlors of five dairy farms in Shandong Province, and bacteria were isolated and identified by mass spectrometry. The reason for selecting the Shandong region for the experiment was that livestock farming in Shandong Province started earlier and lacked rational planning, resulting in higher farming density, and it is currently undergoing a phase of restructuring and development. To investigate the bacterial resistance situation, different concentrations of antibiotics were set by the broth dilution method to evaluate the resistance of the isolated strains to 10 common antibiotics, and the resistance genes were detected by polymerase chain reaction (PCR) to understand the resistance mechanism of bacteria. In recent years, studies have focused on microbiological investigations of air, raw milk, and feces in dairy farms, while there is a relative gap in the study of microbial species and numbers and their antibiotic resistance on floors and utensils that can contact cows and farmers physically in specific places. This study aims to facilitate the selection of antibiotics in dairy farms, to help control the spread of drug-resistant and pathogenic bacteria in the environment, and to provide data references for the promotion of intensive dairy farming to prevent dairy cattle diseases and control drug-resistant bacteria.

## 2. Materials and Methods

### 2.1. Sample Collection

Environmental samples were collected from April to June 2022 in Shandong Province, China. The five farms are two in Qingdao, two in Dongying, and one in Tai’an. Each diry farm conducts ground and equipment sampling in three areas, namely the beddings, milking parlors, and sports fields, with 10 samples taken from each area. 30 samples were collected from each diry farm. A total of 150 environmental samples were collected in this experiment. All the samples were collected in sterile plastic tubes, transported on ice and analyzed within 4 h. 

### 2.2. Isolation and Identification of Environmental Bacteria

Draw 10 μL of bacterial suspension from a centrifuge tube containing the bacterial solution, and dilute it 1000-fold with 0.9% sterile saline (the same procedure is performed for all samples). Collect 5 μL of diluted bacterial suspension and spread evenly on a brain heart infusion (BHI) agar plate (Beijing Land Bridge Technology Co., Ltd., Beijing, China). Incubate the plate at 37 °C in a constant-temperature biochemical incubator for 24 to 48 h. After visible, non-touching single colonies have grown on the plate, observe the morphological characteristics of the colonies and use an inoculation loop to pick a single colony and streak it on a new plate. Incubate the plate at 37 °C for 24 to 48 h, and repeat these steps until only one type of colony is visible. Use an inoculation loop to pick a single colony and place it into a centrifuge tube containing broth BHI liquid medium. Incubate the tube in a biochemical incubator for a period of time, add 20% glycerol, mix thoroughly, and store at −80 °C for future use.

In this study, bacterial species were identified using a mass spectrometer (MALDI-TOF-MS, Bruker Daltonik, Bremen, Germany). The target plate was cleaned with alcohol and wiped clean with a dust-free cloth. Trifluoroacetic acid was then added to clean the target plate, and it was wiped clean with a target plate paper. After the target plate has dried, pick a single colony with a toothpick, and apply it to the metal target plate, add formic acid dropwise, and let it dry naturally. After drying the target plate, add the matrix solution and wait for natural drying to form the co-crystallisation of the matrix and sample. Then the target plate is into MALDI-TOF MS (Bruker Daltonik, Bremen, Germany), the laser is used as the energy source to radiate the crystals, and the sample ions fly to the detector, by analysing the ions with different mass-to-charge ratios (*m*/*z*) to form a spectrum, and compared with the database, to get the identification results and finally determine the bacterial species. 

The picks, centrifuge tubes, and cotton swabs used for sample collection have undergone sterilization via high temperature and pressure. The sterilization process involves maintaining a temperature between 121–126 °C for 30 min, followed by drying in a sterile environment before they are utilized for sample collection.

### 2.3. Antimicrobial Susceptibility Testing

Of the 5 dairy farms examined from the 3 cities studied, it was possible to isolate and identify pathogens in 150 samples, and these were subjected to antibiogram tests. The isolates were tested as described by the Clinical and Laboratory Standards Institute (CLSI)’s operating directions (CLSI M100-S30) to determine their susceptibility to 10 different antimicrobials. The antibiotics tested were 0.16–320 μg/mL ampicillin (PCN), ceftriaxone sodium (CRO), colistin sulphate (CLS), doxycycline (DOX), gentamicin (GM), levofloxacin (LVX), lincomycin (LIN), neomycin (NEO), streptomycin (SM), sulfadiazine (SDZ). The breakpoints of each antibiotic are indicated in parentheses according to the CLSI document M100-S30. The minimum inhibitory concentration tests were performed with the brain heart infusion (BHI, Land Bridge Technology) in 96-well plates (Costa), at 37 °C for 18–24 h. Because some types of bacteria are not recorded in CLSI2020, the sample size of each bacterium in the figure differs for each antibiotic sensitivity.

### 2.4. Detections of Resistance Genes

Single colonies were picked and placed in centrifuge tubes with BHI liquid medium and cultured at 37 °C for some time to promote their multiplication. The cultures were centrifuged at 12,000 rpm for 3 min and resuspended in 1 mL double distilled water (ddH_2_O) and repeated twice, retaining the precipitate. Resuspend the precipitate with 100 µL of ddH_2_O and the suspensions were boiled in a water bath at 100 °C for 10 min. Then the tubes were placed on ice immediately for 10 min, and centrifuged at 13,000 rpm for 5 min to obtain the DNA of each strain. All the extracted DNA was stored at −20 °C.

The reaction system was configured using the primers in Table 1, and after amplification by PCR, the DNA was separated by agarose gel electrophoresis to detect nine ARGs in the strains, including *bla_NDM-1_*, *bla_kpc_* [19], *lnuB*, *tetM*, *ant(4′)-Ia*, *aph(2″)-Ic* [20], *sul1* [21], *qnrS* [22], *mcr-1* [23]. After electrophoresis, the gel plates were placed in a gel imager (Azure Biosystems, Dublin, CA, USA) and the results were visualized and recorded.

### 2.5. Statistical Analysis

To assess the statistical differences in the proportions of antibiotic-resistant strains among the various sampling environments, we employed Fisher’s test. The graphs were made using Origin (2022). Data organization in Excel 2019.

## 3. Results

### 3.1. Isolation and Identification of Bacteria in the Dairy Environment

**Figure 1:**
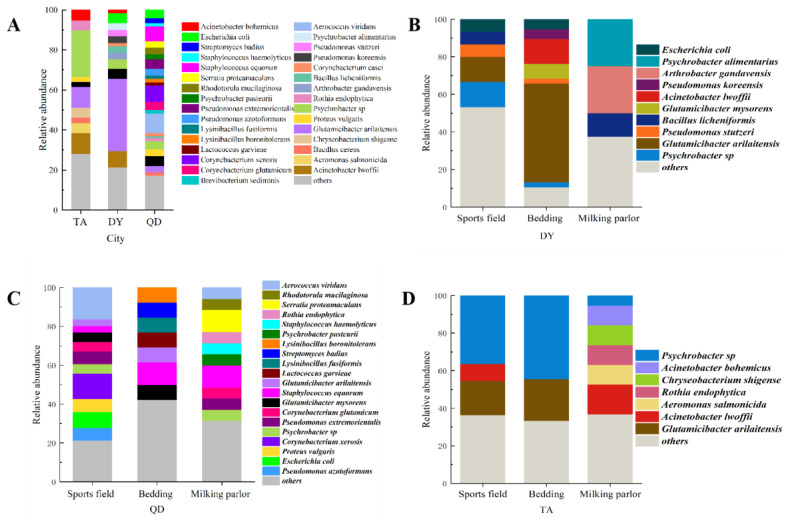
The relative abundance of bacteria in a part of Shandong Province. Relative abundance and relationship of bacteria in dairy farms in (**A**) three cities, (**B**) DY, (**C**) QD, and (**D**) TA.

From Figure 1, the samples were collected from 5 dairy farms, including 50 samples in bedding, 50 samples in sports fields, and 50 samples in milking parlors. A total of 223 bacterial strains were isolated, of which 61 were from Dongying (DY), 122 were from Qingdao (QD), and 61 were from Tai’an (TA).

Sixty-two of the 223 isolates were reported to be pathogenic in the literature, accounting for 27.80% of the total. The relative proportions of isolated environmental pathogens were 20.97% for *Aerococcus viridans* (*n* = 13), 16.13% for *Corynebacterium xerosis* (*n* = 10), 14.52% for *Acinetobacter lwoffii* (*n* = 9), 12.90% for *E. coli* (*n* = 8), 8.06% for *Proteus vulgaris* (*n* = 5), 4.84% for *Bacillus cereus* (*n* = 3), and 22.58% for other bacterial species.

**Figure 2:**
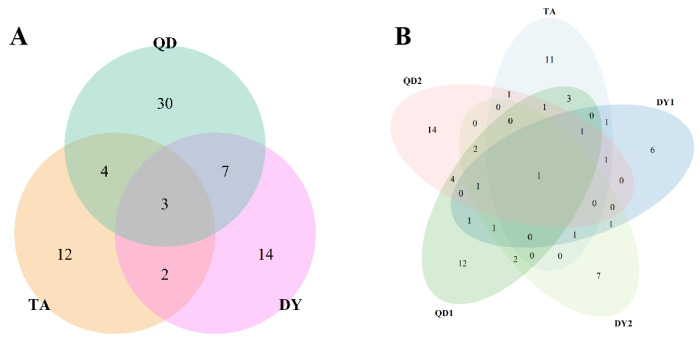
Bacterial correlations across regions and across farms. (**A**) The overlap of bacterial species in the three cities, QD, TA, and DY. (**B**) The overlap of bacterial species in five dairy farms.

Three species of bacteria were jointly isolated in TA, DY, and QD, namely *Psychrobacter* sp., *Glutamicibacter arilaitensis,* and *Glutamicibacter mysorens* (Figure 2). Only *Glutamicibacter arilaitensis* existed in all 5 dairy farms, and the strains isolated from each dairy farm were very different, and there was no obvious correlation.

### 3.2. Antimicrobial Susceptibility Tests of Isolated Strains

According to the micro-broth dilution method recommended by CLSI2020, the susceptibility of 223 strains of bacteria to 10 antibiotics in 7 categories was tested. Since some types of bacteria were not recorded in CLSI2020, each bacterium has a different susceptibility to different antibiotics, and its sample size varies.

**Figure 3:**
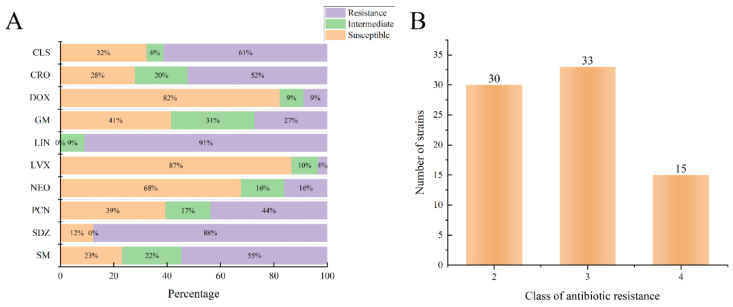
Environmental Bacterial Resistance to Multiple Antimicrobials and the Population of Multidrug-Resistant Bacteria. (**A**) Isolates susceptible to multiple antibiotics; (**B**) number of bacteria strains resistant to three or more types of antibiotics. CLS: Colistin sulfate (*n* = 31); CRO: Ceftriaxone Sodium (*n* = 111); DOX: Doxycycline (*n* = 112); GM: Gentamicin (*n* = 99); LIN: Lincomycin (*n* = 22); LVX: Levofloxacin (*n* = 112); NEO: Neomycin (*n* = 99); PCN: Ampicillin (*n* = 112); SDZ: Sulfadiazine (*n* = 81); SM: Streptomycin (*n* = 99).

It can be seen from Figure 3, that the highest proportions of susceptible isolates were observed for levofloxacin (87%), followed by doxycycline (82%) and neomycin (68%). The lowest proportions of resistance isolates were noted for levofloxacin (4%) and doxycycline (9%). The remaining resistance ratios were observed for Lincomycin (91%), sulfadiazine (88%), colistin sulfate (61%), streptomycin (55%), ceftriaxone sodium (52%) and gentamicin (27%).

A total of 48 multidrug-resistant bacteria were isolated from 5 dairy farms, accounting for 21.52% of the total isolates. The highest proportions of multidrug-resistant bacteria were *Staphylococcus equorum* (*S. equorum*, *n* = 8), followed by *Escherichia coli* (*E. coli*, *n* = 6) and *Proteus vulgaris* (*n* = 5).

### 3.3. Detection of Antibiotic Resistance Genes

By analyzing and summarizing all isolated strains, 62 strains of pathogenic bacteria with literature records were selected from 223 strains for drug resistance gene detection, and some bacteria were not displayed due to insufficient sample size (*n* < 3).

Through the detection of nine kinds of drug resistance genes in 62 strains of pathogenic bacteria, the results are shown in Table 2. *Acinetobacter lwoffii* detected five kinds of ARGs, of which one strain detected the *NDM-1* gene, and more than half of the strains detected *ant(4′)-Ia*, *sul1*, and *qnrS*. A total of seven bacterial isolates containing more than two ARGs were detected.

**Figure 4:**
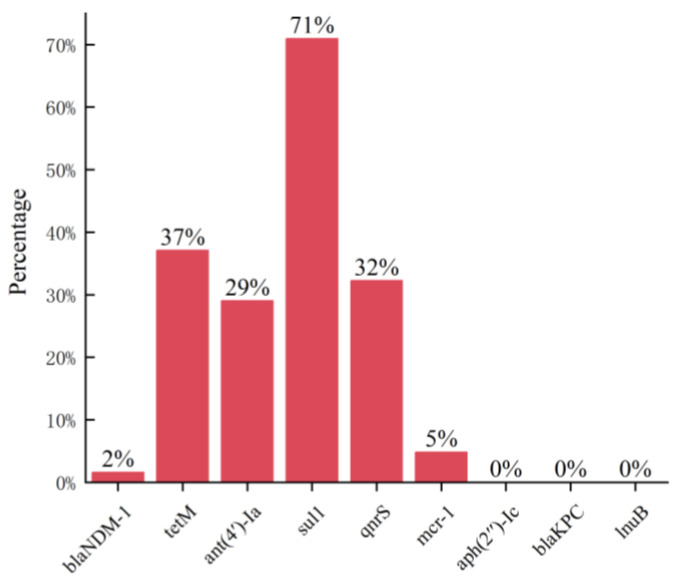
Antibiotic resistance gene detection rate.

By summarizing the ARGs of 62 strains of pathogenic bacteria, the detection rate of the sulfa antibiotic resistance gene *sul1* was the highest, reaching 71%, which was consistent with the performance of sulfadiazine resistance. Aminoglycosides *aph(2′)-Ic*, lincosamides *lnuB*, and β-lactams *bla_kpc_* were not detected (Figure 4).

## 4. Discussion

Since 1942, when penicillin was mass-produced by American pharmaceutical companies and put into use, hundreds of antibiotics have been synthesized to treat infections in humans and animals. There have been reports indicating that China’s agricultural practices have transitioned from traditional free-range farming to intensive livestock production, consequently making it the largest global producer and consumer of antibiotics. Currently, antibiotics are extensively utilized not only in the medical domain but also in sub-therapeutic doses for prolonged periods in fields such as agriculture, forestry, animal husbandry, and fisheries on a global scale. However, the abuse and misuse of antibiotics have led to their excessive overdose being detected many times in surface water, groundwater, and soil worldwide [24,25]. In farming activities, some small and medium-sized farmers have poor awareness of standardized medication use, and the abuse of antibiotics exists more often. When the farm finds that cows are sick, using antibiotic drugs without scientific laboratory diagnosis to determine the cause of the disease, antibiotics kill harmful bacteria while also damaging some beneficial flora, resulting in an imbalance of the bacterial flora in the cow’s body, which triggers the organism disorder, easily causes a decline in the cow’s immunity, and may result in a secondary infection. Dairy farms use a lot of antibiotics in the process of disease prevention and treatment and do not strictly implement the rest period of the drug, which leads to a large number of antibiotic residues in the body of the cow, affecting the growth of the cow as well as triggering the safety hazards of meat products [26]. In the past, the emergence of antibiotics has made it possible to effectively treat many bacterial diseases, but as antibiotics are used more and more frequently, some bacteria have begun to mutate into drug-resistant superbugs, and the emergence of “superbugs” has posed a serious threat to human health. The emergence of antibiotic-induced resistant bacteria is occurring at a much faster rate than the development of new drugs. In recent years, as dairy farming densities have increased, new types of diseases have been rapidly emerging, most of which can infect both humans and animals. So, there is a need to test for bacterial resistance in the dairy farm environment and to develop the right drug regimen based on the situation.

To deepen our understanding of the ecology and bacterial diversity of dairy farms, we isolated and purified bacteria from environmental samples from dairy farms in three cities in Shandong Province, and 223 strains of bacteria were isolated and identified from 150 environmental samples. The three main areas where dairy cows move around the farm are bedding, sports fields, and milking parlors, so environmental hygiene in these areas is a high priority. The results showed that the microbial communities in the three areas of the same dairy farm were significantly different. At the same time, the bacteria isolated from the three cities also differed significantly, with the detection rate of Gram-positive bacteria being higher than that of Gram-negative bacteria. The most representative pathogenic bacteria observed in this study were *Aerococcus viridans*, *Escherichia coli*, *Corynebacterium desiccatum,* and *Fusobacterium lwoffi*. The first two bacteria are common causes of mastitis in dairy cows and have been associated with several diseases in humans [27,28,29]. These pathogens are categorized as environmental pathogens, may contribute to environmental contamination of grazing systems, and are significant risk factors for dairy cows and feeders in the farming process.

The antibiotic resistance test is an important reference indexes for the rational application of antimicrobial drugs in the clinic, and the test enables frontline veterinary workers to make a more reasonable judgment on the clinical use of drugs. In this study, we used the broth dilution method to set different concentrations of antibiotics and determined the resistance of bacteria by observing the growth conditions. From the experimental results of this study, most of the bacteria were resistant to sulfadiazine, and the bacterial isolates from the three urban dairy farm environments were highly resistant to sulfadiazine, both above 80%, while the resistance to levofloxacin and doxycycline was low, below 13%. The resistance to some of the drugs varied from region to region, and we speculate that it is related to the usual medication habits of the farms. Therefore, we suggest that these three cities should avoid repeated use of sulfadiazine and switch to doxycycline and levofloxacin, which have higher sensitivity, while also paying attention to the rational use of drugs to avoid the emergence of multidrug-resistant bacteria.

Antibiotic resistance can develop in different ways, mainly classified into two aspects: on the one hand, antibiotic failure due to the absence or presence of certain structures, which is the natural intrinsic resistance of bacteria; on the other hand, bacteria can develop acquired resistance through chromosomal gene mutations or due to horizontal gene transfer of chromosomes and plasmids, which leads to antibiotic resistance. Residual antibiotics in the environment stimulate the rapid development of ARBs and ARGs through mutation and horizontal gene transfer mechanisms. The presence of ARGs is the main reason for the existence of bacterial resistance [30,31]. In the present study, the highest positivity of the sulphonamide resistance gene *sul1* was found in the bacteria, which is in accordance with the phenotype of high resistance to sulphadiazine. Also, the aminoglycoside resistance gene *aph(2’)-Ic* was not detected in the experiment, which is consistent with the high susceptibility of bacteria to neomycin. However, the resistance genotypes and phenotypes of most of the bacterial strains in this experiment were not uniform, which may be due to the existence of other resistance genes affecting the phenotypes or the unidentified resistance mechanisms, etc. Detecting antibiotic resistance genes carried by bacteria in the environment, controlling the transmission of resistant and pathogenic bacteria, strengthening research on antibiotic resistance genes, reducing the growth of bacterial antibiotic resistance, ensuring the health and welfare of cows, promoting intensive dairy farming, and preventing cattle diseases are of great significance.

## 5. Conclusions

In this study, a total of 223 bacterial strains were isolated from five dairy farms situated in three cities within Shandong Province. Among these farms, the Qingdao Dairy Farm exhibited the highest rate of bacterial isolation, with a relatively elevated presence of pathogenic bacteria. The most frequently encountered bacteria were Fusobacterium and Escherichia. Notably, the isolated bacteria exhibited robust resistance to sulfonamide, with the *sul1* gene, which is closely associated with sulfadiazine resistance, being detected at the highest rate. However, no significant correlation was observed between other antimicrobial resistance genes and antibiotic resistance. Such findings underscore the pressing need to prioritize efforts aimed at enhancing the hygiene conditions prevalent in local pastures.

## Figures and Tables

**Table 1:** Sequences of primers used for detection of antibiotic resistance genes.

gene	5′-3′, F/R	bp
*bla_NDM-1_*	GTCTGGCAGACTTCCTATCTC GGTTCGACAACGCATTGGCATAAG	268
*bla_kpc_*	CGTCTAGTTCTGCTGTCTTG CTTGTCATCCTTGTTAGGCG	798
*lnuB*	CCTACCTATTGTTTGTGGAA ATAACGTTACTCTCCTATTC	925
*tetM*	GTGGACAAAGGTACAACGAG CGGTAAAGTTCGTCACACAC	406
*ant(4′)-Ia*	CAAACTGCTAAATCGGTAGAAGCC GGAAAGTTGACCAGACATTACGAACT	294
*aph(2″)-Ic*	CCACAATGATAATGACTCAGTTCCC CCACAGCTTCCGATAGCAAGAG	444
*sul1*	TTCGGCATTCTGAATCTCAC ATGATCTAACCCTCGGTCTC	822
*qnrS*	ACGACATTCGTCAACTGGAA TTAATTGGCACCCTGTAGGC	417
*mcr-1*	CGGTCAGTCCGTTTGTTC CTTGGTCGGTCTGTAGGG	309

F: forward primer, R: reverse primer, and bp: the number of base pairs.

**Table 2:** Antibiotic resistance gene detection of pathogenic bacteria.

Species		*blaNDM-1*		*tetM*		*ant(4′)-Ia*		*sul1*		*qnrS*		*mcr-1*	
	*n*	*n*	(%)	*n*	(%)	*n*	(%)	*n*	(%)	*n*	(%)	*n*	(%)
*Acinetobacter lwoffii*	9	1	11.11	1	11.11	6	66.67	6	66.67	5	55.56	0	0.00
*Escherichia coli*	8	0	0.00	0	0.00	2	25.00	1	12.50	6	75.00	0	0.00
*Proteus vulgaris*	5	0	0.00	0	0.00	4	80.00	4	80.00	4	80.00	0	0.00
*Corynebacterium xerosis*	9	0	0.00	7	77.78	0	0.00	8	88.89	0	0.00	0	0.00
*Aerococcus viridans*	13	0	0.00	8	61.54	0	0.00	7	53.85	1	11.11	3	33.33
*Bacillus cereus*	3	0	0.00	0	0.00	2	75.00	2	75.00	2	75.00	0	0.00

## Data Availability

Data are contained within the article.
